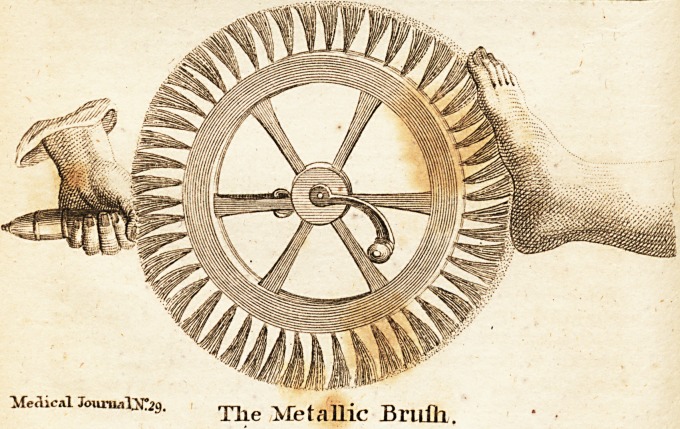# Medical and Physical Intelligence

**Published:** 1801-07

**Authors:** 


Printed for R. Phillips N°. 71. St. Paul's Church Yard.

### MEDICAL AND PHYSICAL INTELLIGENCE.

### [ FOREIGN AND DOMESTIC. ]

*Mr. Molwitz*, of Stutgart, defcribes a new inftrument, which he calls the Metallic Brufh, and from which, nearly the fame effects may be expected as from Perkins's metallic tractors. He is however of opinion, that the action of both inftruments is merely mechanical, but he particularly recommends his Metallic Brufh, as uniting all the different effects of mechanical ftimulufes, as that of friction, preffure, flagellation, brufhing, &c. This inftrument confifts of a fmall wheel, about one foot in diameter, made of any light wood, which has on its right a handle for turning it, and on its left a wooden handle for the left hand, at which it moves through the medium of an iron axle. On its periphery, which is about one inch and a half broad, are faftened bunches of wire, two or three inches long, the top of which end in fmall knobs, like the heads of pins. Nothing material confifts in the fize of the knobs, or the length of the wire, nor in the direction of the bunches. *(See the plate.)* This cheap and fimple inftrument may be applied either by the patient himfelf in fome places, or by fomebody elfe, without requiring any particular dexterity, and it is faid to afford the following advantages. 1. During the operation the inftrument as well as the part on which it is applied can be moved in different directions, and each place touched in fuch a manner as anfwers the purpofe. 2. According to fome obfervations, the application of it on the furface of the belly, feems to promote the periftaltic motion, and to excite the ftomach and bowels to digeftion, and extraction of the chyle, and likewife to carry off wind, and forward the excretion of fæces. 3. The infenfible perfpiration is greatly promoted by it, and on that account its application on the whole furface of the fkin may prove very ufeful in difeafes that arife from a fuppreffed tranfpiration. Mr. Molwitz knew an inftance of a retrograde eryfipelas being reproduced on the fkin by the ufe of the Metallic Brufh. He alfo relates another cafe, where an aged man, who fuffered feverely by rheumatic pains on feveral parts of the body, was cured by four applications of that inftrument. As the patient was fo extremely fenfible at the affected places, it could at the firft time be only applied in circular motions round the affected parts; but the fecond time he could bear the immediate touch of the inftrument on the fuffering parts. 4. This operation feems to have a confiderable influence on animal heat, and the circulation of the blood, as it caufes a quicker pulfe, and a congeftion from the whole fyftem of blood veffels towards the parts that are touched with it. 5. The abforption is thereby con-

ſiderably ſupported and increaſed. Mr. Molwitz relates an inſtance of a conſiderable extravaſation of blood, occaſioned by a fall, being removed by it in a very ſhort time, without the application of any other remedy. 6. Mr. Molwitz thinks, that it might likewiſe be of great uſe in obſtructions and ſpaſms of the belly, cauſed by a ſedentary life, and he mentions a caſe of a painful ſpaſmodic ſenſation in the ſtomach of a woman being cured by it, when the patient could not bear any internal medicine. The operation ought to begin gently, and the inſtrument firſt conducted round the affected part, approaching by degrees towards it; and laſtly, it is not to be applied too long upon the ſame part.

---

The ſame gentleman recommends the topical application of the vapours of hepatic gas, as of great ſervice in arthritic pains ariſing from the continued uſe of mercury, a diſeaſe called by the appropriate name of mercurial gout. The affected foot is accordingly put upon a ſmall bench placed in a bathing tub, into which ſeveral pints of water are poured upon one or two ounces of liver of ſulphur, freſh prepared with lime. The patient having brought the affected part into a convenient poſition, ſeveral glaſſes of ſtrong vinegar are added to the mixture, after which the bathing tub muſt be cloſely covered, that the hepatic gas, which is now diſengaged, may only touch the affected parts; care ought at the ſame time to be taken to ſhelter the mouth and noſe againſt it. He likewiſe praiſes the uſe of the vapours of formic acid, or acid of ants, as being of great ſervice in gouty pains and ſtiffneſs; for which purpoſe any quantity of the great ſpecies of ants is to be infuſed with hot water. Mr. Hufeland adds, that he alſo found in his practice thoſe baths of ants extremely ſerviceable in the gout, and particularly in the worſt kind of it, in the arthritic lameneſs and nodous gout.

---

Dr. Herder, of Weymar, has made ſeveral experiments on the internal uſe of phoſphoric acid, according to which it ſeems to derive its efficacy from acting, not only as a ſtimulus upon excitability, but alſo from imparting to organization, a principle eſſentially neceſſary for its exiſtence. It proved particularly uſeful and beneficial in aſthenic hæmorrhages, conſumptions, diſeaſes of the bones, nervous diſorders, convulſions, lipothymies, &c. Dr. H. was particularly intereſted to diſcover what effect it had in febrile affections; and the experiments he made for the purpoſe convinced him of its being a moſt excellent febrifuge remedy, particularly in nervous fevers with direct debility. In hectic fevers, internal exulcerations, &c. it ſhows itſelf as a very good palliative remedy, by reſtoring the loſs of phoſphoric acid, which the body ſuſtains by profuſe ſweating. His mode of preparing the phoſphoric acid is to burn one drachm of phoſphorus upon a ſtone plate, and to filter the remaining acid with one ounce of diſtilled water. Of this pure diluted acid he adminiſters 15, 20, or 30 drops *pro doſi*, by itſelf, or in combination with a ſyrup. The doſe ought in the beginning beginning to be small, but given at short intervals, and afterwards the dose may be increased, but not employed so frequently.

---

Dr. SAUTER has related, in Hufeland's Practical Journal, Vol. XI. No. 1. some cases of Hydrophobia, occasioned by the bite of a mad dog, in which he successfully employed the belladonna, a remedy that has for these twenty years been known as one of the most efficacious antidotes against that dreadful affection, though in later times the great effect ascribed to it has been much doubted. This Dr. SAUTER thinks is owing to the following circumstances: 1. The belladonna ought always to be given in a sufficient dose, if any decisive effect be expected; because a strong dose at once is better than smaller ones repeatedly given. Dr. SAUTER gave it from ten to twelve grains pro dosi. 2. The root is preferable to the herb of the plant, as being more efficacious. 3. According to the Doctor, the hydrophobia keeps regular periods of forty-eight hours, which ought to be regarded in employing that active and powerful remedy, and, on that account, it should be given in a sufficient dose, in order to prevent the next paroxysm, without losing time by trying other remedies: The best method is, to administer it half an hour or an hour before the second and last paroxysm. With the third paroxysm, the disease proved always mortal, according to the observation of Dr. SAUTER. It is worthy the attention of practitioners, whether the periodical nature of that affection be confirmed by farther experience.

---

Dr. RITTER, of Wisbaden, recommends the application of living snails on obstinate exulcerated buboes, as a sure remedy to heal them. In one case he was obliged to take them off, as the patient could not bear the disagreeable sensation occasioned by the motion of the animal; and for the sake of experiment, he applied the juice of the snails, squeezed in a mortar and expressed: He found that it answered the purpose quite as well as if the living animal had been used. This remedy has been likewise highly praised in scrophulous ulcers, which resisted every other mode of treatment. By what principle they act in such a manner, our theorists have not yet determined.

---

Prof. PLOUCQUET has related a case, in Loder's Journal, by which he endeavours to prove, that in drowned people the wind-pipe and its branches are sometimes entirely filled with water, and as long as this mechanical impediment of breathing exists, no other exciting remedy can be of avail, for bringing the person to life again. As it is impossible to ascertain whether there is water in the trachea or not, it is always advisable and necessary to try to remove that impediment. For this purpose, after the mouth and nose have been sufficiently cleaned, the body ought to be put in such a position, that the head may hang a little downwards, to which end he proposes to lay it across a barrel, or across the back of a strong man, who might at the same time compress the breast and belly, belly, and by that means promote the efflux of the water from the wind-pipe. If, however, this experiment ſhould not ſucceed, he propoſes to extract the water by a ſucking inſtrument, as has been deſcribed by *Van Marum* and *Goodwyn*. For removing the mucous matter in the wind-pipe, he adviſes to inject warm water, either through the rima glottidis, or through an artificial opening. Mr. Plouquet intends to publiſh a treatiſe upon this ſubject, in which he will conſider it more at large.

---

Dr. FLACHSLAND relates a curious inſtance of monſtroſity, which is the more remarkable, as the ſame woman had been delivered three times of children that in every reſpect ſhowed in their ſtructure the ſame deviation from Nature. The face, breaſt, and belly were naturally formed; but the ſuperior extremities meaſured only 3 ½ inches, and the right of the inferior extremities 4, the left 3 ½ inches; they were, at the ſame time, bent inward in ſuch a manner that the heels were near the genitals. The fore arm, on both ſides, was entirely wanting, the humerus being united with the hand by two ligaments; but this union was not effected by a joint, as the condyle of the humerus and the ſuperior margin of the carpus conſiſted of a gelatinous ſubſtance. The tibia and fibula were likewiſe wanting on the inferior extremities, and the feet joined to the patella, in a ſimilar way as was obſerved on the ſuperior extremities. All the inteſtines were naturally formed, and in a natural ſituation.

---

Prof. HECKER, of Erfurt, has related in Hufeland's Practical Journal, two caſes of a diſeaſe which reſembled both the Aſthma Millari and Angina Polypoſa, with the former of which it agreed in being occaſioned by taking cold, and in ſhowing at firſt the ſymptoms of a ſimple cold, which grew worſe by degrees. The noiſe in coughing was a deep baſe, and the paroxyſms of ſuffocation changed in turns with perceptible intermiſſions, which in the beginning ſeldom, but afterwards rapidly ſucceeded one another, and death followed from a ſpaſmodic ſuffocation. It reſembled Angina Polypoſa in owing its origin to an infectious miaſma, in the violent coughing which accompanied it, and particularly in the polypous compact concretions that were found in the wind pipes of ſome patients who died of this diſeaſe. Mr. Hecker therefore calls it Angina Polypoſa ſpaſmodica, as it is partly occaſioned by a ſpaſtic coarctation and contraction of the pulmonary veſſels, and partly by polypous concretions in the trachea and its branches.— The principal remedy which he ſucceſsfully employed, was mercury in combination with extractum hyoſciami, or, if circumſtances would permit it, with opium. It ought, however, to be given in ſuch doſes as to be capable of acting inſtantly upon the conſtitution, and the ſymptoms of its action muſt be ſoon perceived in the mouth by a gentle ſalivation. The addition of narcotics prevents its acting upon the bowels. It is ſometimes neceſſary and of much

ſervice fervice to adminifter an emetic during the ufe of mercury, in order to caufe the patient to vomit up the concretions that have been made removeable by the mercury, and at the fame time to promote the ceffation of the fpafms in the lungs. External mercurial frictions on the affected part are alfo ufeful for diffolving the polypous concretions; and a mixture of unguentum mercurii and linimentum volatile proved very efficacious in thefe cafes. Mr. Hecker finally remarks, that not any good is to be expected from tracheotomy, as the proximate caufe of death is not owing to a mere fpaftic and mechanic conftriction of the rima glottidis, but to the trachea and its branches being filled and obftructed with coagulated lymph.

---

According to the obfervations of Mr. Stern, Semina Phellandrii Aquatici, deferve very much to be recommended in confumptions. Its efficacy, he thinks, is particularly owing to its promoting the clean ftate of the pulmonary ulcer. He generally employed it with Pulvis Doveri, chiefly in order to diminifh the inflammatory irritation of the ulcer, and to render the cough lefs frequent. The nature of the pus ought always to be obferved, becaufe if it is in a too thick or putrid ftate, it cannot well be evacuated by urine, and the remedy becomes therefore lefs ufeful. Mr. Stern is of opinion, that it may likewife prove a very efficacious remedy in hooping cough; but this requires to be afcertained by farther experiments. The feeds of Phellandrium Aquaticum have alfo a vermifuge quality.

---

Dr. Jenner has communicated in a letter to Dr. De Carro of Vienna, his experiments on inoculating dogs with Cow-pox, which produced in them a difeafe, fimilar to that which is commonly called the dog's diftemper, but in a very flight degree. What is moft remarkable, this inoculation renders them afterwards unfufceptible of that affection. Dr. De Carro has hitherto had no opportunity of repeating thofe experiments on a large fcale; but the few he has made, perfectly agreed with what Dr. Jenner had obferved.

---

To Dr. BRADLEY,

Dear Sir,

In a letter from a friend of mine at Palermo, dated the 21ft of April, is the following paffage, which I extract, becaufe you may think it worth a place in your next number, under the article of Medical News. Your's, always,

*New Burlington Street, June 15, 1801.* JOHN CLARKE.

"The Cow-pox is in high reputation at Palermo. I am well acquainted with Dr. Marfhall, who tells me that he has tried it already upon nearly 10,000 perfons, and it has generally fucceeded better than in colder climates. His fees for inoculating the Cow-pox, are ten guineas in genteel families, and five in families of the the middle claſs. The poor he inoculates gratuitouſly. The king has deſired the prieſts to recommend it from the pulpit, and ſeems much intereſted in the ſucceſs of the practice, and not without reaſon, as 8000 perſons died of the Small-pox alone laſt year in Palermo. Sicily only wants population to make it rival many European nations, and this practice is ſurely a very effectual means of providing for that deficiency. It is intended to convey the diſeaſe to Turkey, and the Engliſh will have the credit of ſending thither a remedy, by means of which the Small-pox may in time be eradicated."

---

Mr. Medley, Portrait Painter, has iſſued propoſals for publiſhing by ſubſcription a print of the Medical Society of London, containing twenty-two portraits, from the picture painted by him, now engraving by N. Branwhite; ſize of the plate 22 ¼ inches by 18 ½ inches; price to ſubſcribers for early impreſſions, two guineas each, common impreſſions 1l. 11s. 6d.

The portraits are of the following medical gentlemen,

| | | |
|---|---|---|
| Dr. Sims | Dr. Walker | |
| Sir John Hayes | Dr. Thornton | |
| Dr. Lettſom | Dr. Shadwell | |
| Dr. Hulme | Dr. Haighton | |
| Dr. Saunders | Dr. Hooper | |
| Dr. Relph | Dr. Jenner | |
| Dr. Combe | Mr. Fo d | Surgeons |
| Dr. Woodville | Mr. Ware | |
| Dr. Bancroft | Mr. Blair | |
| Dr. Myers | Dr. Aſh | Buſts of deceaſed Members |
| Dr. Bradley | Dr. Smith | |
| Dr. Aikin | Mr. Hewſon | |
| Dr. Babington | | |

Subſcriptions are taken in by S. Medley, No. 52, Thread-needle Street; no money to be taken till the delivery of the print.

N. B. The above print was begun in the month of December, 1799, and is now in ſuch a ſtate of forwardneſs, as to aſſure thoſe who have patronized the undertaking of its ſpeedy publication.

---

Mr. J. R. Smith has juſt publiſhed a fine Print of Dr. Jenner, to whom the world is ſo highly indebted for the introduction of the Cow-pox Inoculation; the print is an excellent likeneſs, and does great credit to the artiſt, who is both the painter and engraver.

## Figures and Tables

**Figure f1:**